# Association of Different Anticoagulation Strategies With Outcomes in Patients Hospitalized With Acute Pulmonary Embolism

**DOI:** 10.7759/cureus.61545

**Published:** 2024-06-02

**Authors:** Abdul Rehman, Jeeyune Bahk, Hafiza Noor U Baloch, Sidra Salman, Venus Sharma, Avinash Singh, David J Steiger

**Affiliations:** 1 Internal Medicine, Rutgers University New Jersey Medical School, Newark, USA; 2 Internal Medicine, Mount Sinai Hospital, New York City, USA; 3 Pulmonary and Critical Care Medicine, Mount Sinai Hospital, New York City, USA

**Keywords:** hemorrhage, anticoagulation bridge therapy, mortality, length of hospital stay, acute pulmonary embolism

## Abstract

Background

Therapeutic anticoagulation is the cornerstone of treatment for pulmonary embolism (PE), but the impact of different anticoagulation strategies on patient outcomes remains unclear. In this study, we assessed the association of different anticoagulation strategies with the outcomes of patients with acute PE.

Methods

A retrospective chart review of 207 patients with acute PE who were admitted to one of three urban teaching hospitals in the Mount Sinai Health System (in New York City) from January 2020 to September 2022 was performed. Demographic, clinical, and radiographic data were recorded for all patients. Multivariate regression analyses were performed to assess the association of different outcomes with the approach of therapeutic anticoagulation used.

Results

The median age of the included patients was 65 years, and 50.2% were women. The most common approach (n = 153, 73.9%) to therapeutic anticoagulation was initial treatment with unfractionated or low molecular weight heparin followed by a direct-acting oral anticoagulant (DOAC), while heparin alone (either unfractionated or low molecular weight heparin) was used in 37 (17.9%) patients, and another 17 (8.2%) patients were treated with heparin followed by bridging to warfarin. Hospital length of stay was longer for patients in the "heparin to warfarin" group (risk-adjusted incidence rate ratio of 2.52). The rates of in-hospital bleeding, all-cause 30-day mortality, and all-cause 30-day re-admissions did not have any significant association with the therapeutic anticoagulation approach used.

Conclusion

Patients with acute PE who were initially treated with heparin and subsequently bridged to warfarin had a longer hospital stay. Rates of in-hospital bleeding, 30-day mortality, and 30-day re-admission were not associated with the strategy of therapeutic anticoagulation employed.

## Introduction

Pulmonary embolism (PE) is the third most common acute cardiovascular disease worldwide after myocardial infarction and stroke [1]. In the United States, the incidence of acute PE was estimated to be 117 per 100,000, although this may well be an underestimate [2]. The mortality rate for acute PE ranges from 6% in low-risk cases to 40% in patients with hemodynamic instability [3]. In the United States, more than USD 1.5 billion is spent annually on managing patients with acute PE, which represents an enormous economic burden on the healthcare system [4].

Therapeutic anticoagulation is the cornerstone of treatment for acute PE [1]. Historically, the most common anticoagulation strategy was the initiation of either low molecular weight heparin (LMWH) or unfractionated heparin (UFH), followed by a subsequent transition to warfarin. The development of direct-acting oral anticoagulants (DOAC) has drastically reduced the use of warfarin and enabled the outpatient treatment of acute PE in selected low-risk patients [5]. Nevertheless, the use of warfarin along with bridging (overlapping) anticoagulation continues due to certain compelling medical indications (such as antiphospholipid antibody syndrome), patient preference, clinician preference, and DOAC availability or affordability issues [6]. Moreover, the initial choice of anticoagulant (UFH versus LMWH) and the timing of transitioning to DOAC among patients receiving catheter-directed or surgical thrombectomy differ widely among centers [7]. The role of catheter-directed interventions in the treatment of patients with intermediate-low-risk and intermediate-high-risk PE is an area of active research. Due to the paucity of randomized controlled trials comparing catheter-directed management versus standard therapeutic anticoagulation [8,9] in patients at increased risk of PE-related mortality, management is based mostly on expert consensus and observational data [10-13]. The management of anticoagulation in such patients is also an area of clinical equipoise.

The impact of different strategies of anticoagulation on hospital LOS and clinical outcomes among patients hospitalized with acute PE remains unclear. We hypothesized that the use of different agents of anticoagulation and the time of transitioning to DOAC or warfarin may affect hospital length of stay (LOS) and may influence the risk of bleeding complications in patients with acute PE. In the present study, we investigated the association of LOS, mortality, and bleeding complications with different strategies of anticoagulation among patients hospitalized with acute PE. We aimed to determine if the difference in anticoagulant use and timing of the transition from a heparin-based anticoagulant to a DOAC or warfarin was associated with changes in hospital LOS, bleeding complications, and mortality.

## Materials and methods

This retrospective observational study included patients treated at three urban teaching hospitals included in the Mount Sinai Health System (viz., Mount Sinai Morningside, Mount Sinai West, and Mount Sinai Beth Israel). The study was approved by the institutional IRB. A consecutive sample of all patients who had PERT (pulmonary embolism response team) activations and were diagnosed with acute PE between January 2020 and September 2022 was used in this study. PE was diagnosed in all patients by visualization of filling defects within pulmonary arteries on thoracic CT angiograms.

PE response team

In the Mount Sinai system of teaching hospitals, a dedicated PE response team (PERT) facilitates the management of all patients who are deemed to have intermediate or high-risk acute PE. A PERT consultation is called for patients with acute PE who either have confirmed or suspected evidence of right heart dysfunction or hemodynamic instability. In some cases of low-risk PE, PERT consultation may also be requested at the discretion of the primary treating clinician. The PERT is multidisciplinary and is comprised of cardiologists, pulmonologists, cardiothoracic surgeons, interventional radiologists, and intensivists. Discussion between members of the PERT leads to a consensus decision for clinical management that is individualized to patient characteristics.

Standardized care

The 2019 guidelines from the European Society of Cardiology (ESC) were used as the reference standard for risk stratification and management of patients with acute PE [14]. All patients were treated with therapeutic anticoagulation if the risk of bleeding was not considered prohibitive. In patients with high-risk PE (hemodynamically unstable), systemic thrombolysis was administered if there were no contraindications. Catheter-directed or surgical embolectomy was also performed for eligible patients if deemed feasible. The FlowTriever® (Inari Medical, Irvine, CA) device was used to perform percutaneous catheter-directed embolectomy by experienced interventional radiologists. A single cardiothoracic surgeon performed a surgical embolectomy through a midline sternotomy approach after a total cardiopulmonary bypass. In patients who were at risk of recurrent PE and had a high risk of bleeding, an inferior vena cava filter was also inserted.

Data collection

All patients who had PERT activation during the study period and had an ICD-10-CM diagnosis code of pulmonary embolism were eligible for inclusion in the study. For all eligible patients, the electronic medical record was systematically reviewed to collect data pertaining to demographics, medical comorbidities, risk factors for PE, vital signs, laboratory investigations, radiographic imaging, echocardiographic findings, LOS, mortality, and bleeding complications. The outcomes assessed in this study included hospital LOS, 30-day mortality, in-hospital clinically relevant bleeding of any severity, and 30-day re-admission. Data was initially recorded on standardized spreadsheets, which were later imported into statistical software for performing statistical analysis.

Therapeutic anticoagulation

Therapeutic anticoagulation regimens for acute PE included unfractionated heparin (UFH), low molecular weight heparin (LMWH), direct-acting oral anticoagulants (DOAC), and warfarin. UFH was administered in the form of a continuous intravenous infusion, which was titrated to achieve an active partial thromboplastin time between 70 and 110 seconds (based on institutional protocol). Active partial thromboplastin time was measured every six hours until two consecutive readings were within the therapeutic range, following which measurement was reduced to every 12 to 24 hours. LMWH was administered subcutaneously in a weight-based dosage of either 1 mg/kg every 12 hours or 1.5 mg/kg every 24 hours. DOACs used at the study sites included apixaban, rivaroxaban, dabigatran, and edoxaban. Warfarin was administered to target an international normalized ratio of 2.0 to 3.0 for most cases (in exceptional cases, a lower or higher range was targeted based on the discretion of the treating clinician). Bridging (overlapping) anticoagulation with either LMWH or UFH was administered to patients being started on warfarin therapy for a minimum duration of three days or until the international normalized ratio was within the therapeutic range (whichever was longer).

Statistical considerations

Descriptive analyses were performed in IBM Corp. Released 2011. IBM SPSS Statistics for Windows, Version 20.0. Armonk, NY: IBM Corp., while multivariate regression analyses were performed in R version 4.1.1 (R Foundation for Statistical Computing, Vienna, Austria). For qualitative variables, frequencies and percentages were calculated. For quantitative variables, mean and standard deviation (SD) were calculated if the variables were normally distributed, or median and interquartile range (IQR) were calculated if the variables did not follow a normal distribution. For assessing the impact of different approaches to anticoagulation on outcomes, the dataset was divided into three groups based on the strategy of anticoagulation employed: (a) UFH or LMWH alone; (b) UFH or LMWH followed by transition to a DOAC; or (c) UFH or LMWH followed by bridging to warfarin. A multivariate regression analysis was performed to assess the impact of different approaches to therapeutic anticoagulation on primary outcomes. For outcomes such as mortality, in-hospital bleeding, and re-admissions, log-binomial regression models were constructed to calculate prevalence (risk) ratios along with 95% confidence intervals (CI). For assessing hospital LOS, a negative binomial regression model was used to calculate incidence rate ratios (IRR) along with the calculation of robust standard errors. A p-value of less than 0.05 was considered statistically significant for all comparisons.

## Results

**Figure 1:**
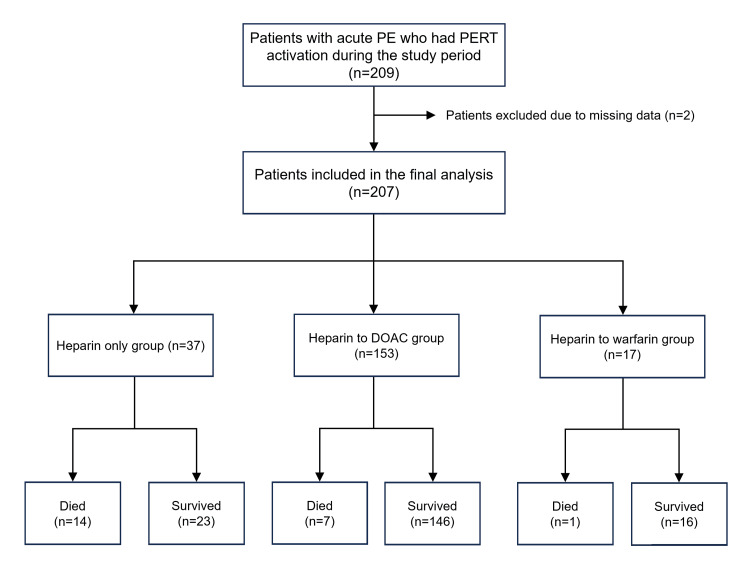
A flowchart of patients included in the study and final analysis. DOAC: Direct-acting oral anticoagulant; PE: pulmonary embolism; PERT: pulmonary embolism response team.

During the study period, 209 patients were diagnosed with acute PE and had PERT consultations. Of these, two patients were excluded due to missing data on the details of anticoagulation. This left a total of 207 patients, which were included in the final analysis (Figure 1).

Demographics and baseline clinical features

The median age of patients included in the study was 65 (IQR: 53-75) years, with an almost equal proportion of men (n=103, 49.8%) and women (n=104, 50.2%). White (n=114, 55.1%) and black (n=85, 41.1%) races comprised the majority of the study patients. Only 21 (10.1%) patients self-identified as Hispanic or Latino. The median body mass index (BMI) of the included patients was 27.6 (IQR: 23.9-34.5) kg/m2, and 40.6% of patients were obese (n=84). The median Charlson Comorbidity Index for included patients was three (1-5) points. Thirty-seven (17.9%) patients had active malignancies, while another 24 (11.6%) were active smokers. A prior history of venous thromboembolism (VTE) was reported by 44 (21.3%) patients, while another 32 (15.5%) had completed a prior course of anticoagulation. Further details of risk factors and the past medical history of the included patients are provided in Table 1.

**Table 1:** Demographic and clinical features for the combined study cohort (n=207) AC: Anticoagulation; BMI: body mass index; BP: blood pressure; CT: computed tomography; DVT: deep venous thrombosis; ESC: European Society of Cardiology; INR:  international normalized ratio; IQR: interquartile range; IVC: inferior vena cava; LV: left ventricle; PASP: pulmonary artery systolic pressure; PE: pulmonary embolism; PESI: Pulmonary Embolism Severity Index; RDW: red blood cell distribution width; RV: right ventricle; SD: standard deviation; VTE: venous thromboembolism. * Lower extremity venous duplex ultrasonography was performed in 158 patients (76.4%) included in the study † Transthoracic echocardiography was performed on 193 patients (93.2%) included in the study ‡ Either intravenous unfractionated heparin drip or subcutaneous low molecular weight heparin

Characteristics	Results
Age (median [IQR])	65 (53 to 75) years
Sex	
Female	104 (50.2%)
Male	103 (49.8%)
Race	
Asian	6 (2.9%)
Black	85 (41.1%)
White	114 (55.1%)
Other	2 (0.9%)
BMI (median [IQR])	27.6 (23.9 to 34.5) kg/m^2^
Charlson Comorbidity Index (median [IQR])	3 (1 to 5) points
Past medical history	
Previous course of AC	32 (15.5%)
Left-sided heart failure	11 (5.3%)
Pulmonary hypertension	4 (1.9%)
Chronic lung disease	36 (17.4%)
Risk factors	
Active malignancy	37 (17.9%)
Active smoking	24 (11.6%)
Immobility for ≥ 3 days	14 (6.8%)
Predisposing medications	10 (4.8%)
Prior history of VTE	44 (21.3%)
Recent long flight	7 (3.4%)
Surgery <4 weeks ago	13 (6.3%)
Clinical features on presentation	
Pulse rate >110/min	60 (29.0%)
Systolic BP <100 mm Hg	14 (6.8%)
Temperature < 96.8°F	12 (5.8%)
Respiratory rate >30/min	4 (1.9%)
Laboratory investigations (median [IQR])	
Blood urea nitrogen	17 (IQR: 11 to 23) mg/dl
Brain natriuretic peptide	145 (IQR: 52 to 342) pg/ml
Creatinine	0.98 (IQR: 0.80 to 1.27) mg/dl
D-dimer	11.8 (IQR: 5.79 to 20) mcg/ml
INR	1.2 (IQR: 1.1 to 1.3)
Platelet count	203 (IQR: 155 to 255) × 10^9^ cells/L
RDW at diagnosis	13.0 (IQR: 12.2 to 14.4)
Troponin I	0.11 (IQR: 0.04 to 0.34) ng/ml
PESI score [mean (SD)]	88.8 (SD: 30.3) points
PESI risk class	
Class I	40 (19.3%)
Class II	64 (30.9%)
Class III	52 (25.1%)
Class IV	28 (13.6%)
Class V	23 (11.1%)
ESC risk class	
Low risk	6 (2.9%)
Intermediate-low risk	50 (24.1%)
Intermediate-high risk	132 (63.8%)
High risk	19 (9.2%)
CT scan findings	
Saddle PE	47 (22.7%)
Central PE	175 (84.5%)
Peripheral PE	128 (61.8%)
Pulmonary infarct	28 (13.5%)
RV enlargement	173 (83.6%)
DVT on lower extremity ultrasonography*	85 (41.1%)
Findings of transthoracic echocardiography†	
PASP (median [IQR])	38 (28 – 50) mm Hg
RV dysfunction	141 (68.1%)
LV systolic dysfunction	33 (15.9%)
LV diastolic dysfunction	25 (12.1%)
Therapeutic interventions	
Systemic thrombolysis	13 (6.3%)
Catheter-directed embolectomy	16 (7.8%)
Surgical embolectomy	39 (18.8%)
IVC filter insertion	34 (16.4%)
Anticoagulation strategy	
Heparin‡ alone	37 (17.9%)
Heparin‡ to DOAC	153 (73.9%)
Heparin‡ to warfarin	17 (8.2%)

Pulmonary embolism was diagnosed by thoracic CT scans in all patients. Saddle, central, and peripheral PE were noted in 47 (22.7%), 175 (84.5%), and 128 (61.8%) CT scans, respectively. Pulmonary infarction was noted in 28 (13.5%) patients, while RV enlargement (on CT scan) was noted in 173 (83.6%) patients. The mean PESI score of the included patients was 88.8 (SD: 30.3) points, and PESI classes I, II, III, IV, and V accounted for 19.3%, 30.9%, 25.1%, 13.6%, and 11.1% of patients, respectively. Based on ESC risk stratification, low-risk, intermediate low-risk, intermediate high-risk, and high-risk PE were noted in six (2.9%), 50 (24.1%), 132 (63.8%), and 19 (9.2%) cases, respectively. Evidence of RV dysfunction by transthoracic echocardiography was noted in 68.1% (n=141) of patients. Further details of clinical features and laboratory investigations are provided in Table 1.

Therapeutic interventions and anticoagulation

Systemic thrombolysis was administered to 13 (6.3%) patients. Surgical embolectomy and catheter-directed embolectomy were performed in 18.8% (n=39) and 7.8% (n=16) of patients, respectively. Another 34 (16.4%) patients required inferior vena cava filter insertion. The median hospital LOS of the included patients was six (3-10) days. The 30-day mortality rate was 10.6% (n=22). Clinically relevant bleeding of any severity occurred in 6.8% (n=14) of patients. Another 23 (11.1%) patients required re-admission within 30 days. Of these, 7/23 (30%) of re-admissions were for VTE, and one re-admission was for bleeding. The remaining re-admissions were due to non-VTE-related causes.

Therapeutic anticoagulation was initially started in all patients included in the study. UFH (n=163, 78.7%) and LMWH (n=42, 20.3%) were the two most commonly used agents for initial anticoagulation. Warfarin (n=1) and apixaban (n=1) were only continued as the initial anticoagulant in one patient each. Upon discharge, the most commonly prescribed anticoagulant was a DOAC (n=153, 73.9%). Warfarin was prescribed as the final anticoagulant to be given on discharge in 17 (8.2%) patients. The reasons for choosing warfarin therapy in these patients were antiphospholipid antibody syndrome (n=5), affordability reasons (n=4), patient preference (n=2), and clinician discretion (n=6). LMWH was prescribed at the time of discharge in the remaining patients (n=37, 17.9%).

Effect of anticoagulation strategy on outcomes

**Figure 2:**
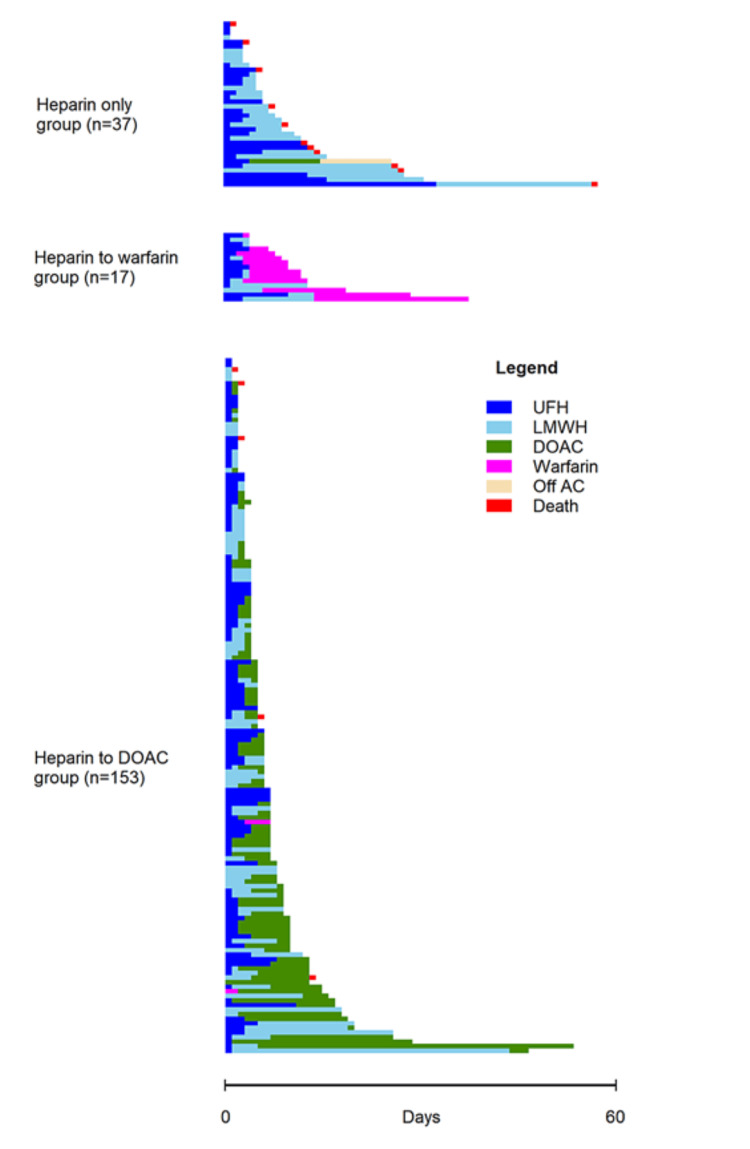
A schematic diagram depicting the duration of different anticoagulant therapies within each group. Note that some patients were discharged from anticoagulation due to clinically significant bleeding. AC : Anticoagulation; DOAC: direct-acting oral anticoagulant; LMWH: low molecular weight heparin; UFH: unfractionated heparin.

For assessing the association between different anticoagulation approaches and specific patient outcomes, we divided the patient cohort into three groups based on the choice of final anticoagulant. The most common anticoagulation strategy employed DOAC as the final anticoagulant of choice (n = 153)-the “heparin to DOAC” group. The second group consisted of patients who were discharged on warfarin (n=17). The remaining patients were included in the “heparin-only” group (n=37). This group included patients who were either discharged on LMWH or died before they could be transitioned to any other anticoagulant. The timeline of different anticoagulants amongst these three groups of patients is depicted in Figure 2. The median duration of in-patient anticoagulant per patient was six (IQR: 4-12) days, five (IQR: 3-8) days, and 10 (IQR: 8-13) days in the “heparin only,” “heparin to DOAC,” and “heparin to warfarin” groups, respectively. The median duration from the initial anticoagulant to final anticoagulant was three (IQR: 2-5) days and four (IQR: 3-4) days in the “heparin to DOAC” and “heparin to warfarin” groups, respectively.

The demographic and baseline characteristics of the three groups are summarized in Table 2. Except for BMI, there were no statistically significant differences between the three groups in terms of baseline characteristics. Patients in the “heparin to warfarin” group had a higher BMI (median BMI: 36.8 [IQR: 28.9-42.7]) compared to other groups. The median hospital LOS was shortest in the “heparin to DOAC” group (at seven [IQR: 5-11] days) and longest in the “heparin to warfarin” group (at 11 [IQR: 9-15] days). Based on univariate analysis (negative binomial regression), the crude IRR (incidence rate ratio) was 2.87 (95% CI: 1.34-4.18) for the “heparin to warfarin” group (Table 3). This association remained significant after adjusting for age, sex, BMI, Charlson Comorbidity Index, PESI score, ESC risk group, and five other confounding variables (adjusted IRR 2.52 [95% CI: 1.12-3.47]). The 30-day mortality rates for the “heparin only," “heparin to DOAC,” and “heparin to warfarin” groups were 37.8% (14 of 37), 4.6% (7 of 153), and 0.1% (1 of 17), respectively (Table 3). Note that the “heparin only” group consisted of all those patients who had died before being switched to either warfarin or DOAC and, therefore, overrepresented patients who died. The risk-adjusted prevalence (risk) ratio for 30-day mortality in the “heparin to DOAC” group was 0.13 (95% CI: 0.05-0.28), which was statistically significant. On the other hand, the risk-adjusted prevalence ratio for the “heparin to warfarin” group did not reach statistical significance, likely owing to the small number of events in this group (Table 3). When an additional analysis was performed by combining the “heparin only” and “heparin to DOAC” groups, the risk-adjusted prevalence ratio was not significantly different for the “heparin to warfarin” group (0.55 [95% CI: 0.02-4.64]). Similarly, after combining the “heparin only” and “heparin to warfarin” groups, the risk-adjusted prevalence ratio was not significantly different for the “heparin to DOAC” group (1.02 [95% CI: 0.23-2.94]). The rates of in-hospital bleeding complications for the “heparin only," “heparin to DOAC,” and “heparin to warfarin” groups were 8.1% (3 of 37), 5.9% (9 of 153), and 11.8% (2 of 17), respectively. There was no significant association between the strategy of anticoagulation and the risk of in-hospital bleeding on multivariate regression analysis (Table 3). Likewise, there was no significant association between the 30-day re-admission rate and the strategy of anticoagulation (Table 3). The 30-day all-cause re-admission rates for the “heparin only," “heparin to DOAC,” and “heparin to warfarin” groups were 18.9% (7 of 37), 9.1% (14 of 153), and 11.8% (2 of 17), respectively. VTE-related causes accounted for six and two re-admissions in the “heparin only” and “heparin to DOAC” groups, respectively. Only one bleeding-related re-admission occurred in the “heparin-only” group. Multivariate regression analyses for cause-specific 30-day re-admission rates were not performed, given the small number of events in each group.

**Table 2:** Risk profile of patients included in different anticoagulation groups BMI: Body mass index; CCI : Charlson Comorbidity Index; CI: confidence interval; CT: computed tomography; DOAC: direct-acting oral anticoagulant; ESC: European Society of Cardiology; PE: pulmonary embolism

Characteristics	Results		
	Heparin only	Heparin to DOAC	Heparin to warfarin
Number of patients	37 (17.9% of sample)	153 (73.9% of sample)	17 (8.2% of sample)
Sex			
Female	21 (56.7%)	76 (49.7%)	7 (41.2%)
Male	16 (43.2%)	77 (50.3%)	10 (58.8%)
Body mass index			
Median BMI (IQR), kg/m^2^	27.3 (22.5 – 33.3)	27.3 (24.0 – 33.9)	36.8 (28.9 – 42.7)
Charlson Comorbidity Index			
Median CCI (IQR)	4 (2 – 7)	3 (1 – 5)	1 (1 –3)
PESI class			
I	7 (18.9%)	26 (17.0%)	7 (41.2%)
II	7 (18.9%)	50 (32.7%)	7 (41.2%)
III	7 (18.9%)	42 (27.4%)	3 (17.6%)
IV	7 (18.9%)	21 (13.7%)	0 (0%)
V	9 (24.4%)	14 (9.2%)	0 (0%)
ESC risk class			
Low-risk	1 (2.7%)	4 (2.6%)	1 (5.9%)
Intermediate low-risk	14 (37.8%)	32 (20.9%)	4 (23.5%)
Intermediate high-risk	15 (40.6%)	106 (69.3%)	11 (64.7%)
High-risk	7 (18.9%)	11 (7.2%)	1 (5.9%)
Where PE was diagnosed			
Emergency department	33 (89.2%)	146 (95.4%)	14 (82.4%)
Inpatient	4 (10.8%)	7 (4.6%)	3 (17.6%)
Incidental PE			
Yes	2 (5.4%)	6 (3.9%)	1 (5.9%)
No	35 (94.6%)	147 (96.1%)	16 (94.1%)
Radiographic findings on CT			
Saddle PE	4 (10.8%)	39 (25.5%)	4 (23.5%)
Central PE	33 (89.2%)	128 (83.7%)	14 (82.4%)
Peripheral PE	27 (73.0%)	94 (61.4%)	7 (41.2%)
Pulmonary infarction	3 (8.1%)	23 (15.0%)	2 (11.8%)
RV dysfunction			
Yes	28 (75.7%)	136 (88.9%)	14 (82.4%)
No	9 (24.3%)	17 (11.1%)	3 (17.6%)
Therapeutic interventions			
Systemic thrombolysis	2 (5.4%)	9 (5.9%)	2 (11.8%)
IVC filter insertion	10 (27.0%)	20 (13.1%)	4 (23.5%)
Catheter-directed embolectomy	3 (8.1%)	11 (7.2%)	2 (11.8%)
Surgical embolectomy	3 (8.1%)	32 (20.9%)	4 (23.5%)
In-hospital bleeding			
Yes	3 (8.1%)	9 (5.9%)	2 (11.8%)
No	34 (91.9%)	144 (94.1%)	15 (88.2%)
Outcome at 30 days			
Died	14 (37.8%)	7 (4.6%)	1 (5.9%)
Survived	23 (62.2%)	146 (95.4%)	16 (94.1%)
Re-admission within 30 days			
Yes	7 (18.9%)	14 (9.2%)	2 (11.8%)
No	30 (81.1%)	139 (90.8%)	15 (88.2%)

**Table 3:** Association of different anticoagulation strategies with length of stay CI: Confidence interval; DOAC: direct-acting oral anticoagulant; LOS: length of stay; IQR: interquartile range; VTE: venous thromboembolism. * Incidence rate ratio computed from a multivariate negative binomial regression model with the calculation of robust standard errors † Adjusted for age, sex, body mass index, Charlson Comorbidity Index, PESI (Pulmonary Embolism Severity Index) score, ESC (European Society of Cardiology) risk class, RDW (red cell distribution width), saddle embolism, need for systemic thrombolysis, need for surgical embolectomy, and need for catheter-directed embolectomy ‡ Prevalence (risk) ratio computed from a multivariate binomial regression model with a log link function

LENGTH OF STAY				
Measure		Heparin only group	Heparin to DOAC group	Heparin to warfarin group
Number		37 (17.9% of sample)	153 (73.9% of sample)	17 (8.2% of sample)
Median LOS (IQR)		9 (5 to 14) days	7 (5 to 11) days	11 (9 to 15) days
Crude incidence rate ratio* (95% CI)		1.0 (Reference group)	0.72 (0.50 to 1.01)	2.87 (1.34 to 4.18)
Risk-adjusted† incidence rate ratio* (95% CI)		1.0 (Reference group)	0.69 (0.80 to 1.02)	2.52 (1.12 to 3.47)
MORTALITY AT 30 DAYS				
Measure		Heparin only group	Heparin to DOAC group	Heparin to warfarin group
Number		37 (17.9% of sample)	153 (73.9% of sample)	17 (8.2% of sample)
Events		14/37 (37.8%)	7/153 (4.6%)	1/17 (5.9%)
Crude prevalence ratio‡ (95% CI)		1.0 (Reference group)	0.07 (0.02 – 0.23)	0.15 (0.01 – 0.68)
Risk-adjusted† prevalence ratio‡ (95% CI)		1.0 (Reference group)	0.13 (0.05 – 0.28)	0.14 (0.05 – 1.19)
IN-HOSPITAL BLEEDING				
Measure		Heparin only group	Heparin to DOAC group	Heparin to warfarin group
Number		37 (17.9% of sample)	153 (73.9% of sample)	17 (8.2% of sample)
Events		3/37 (8.1%)	9/153 (5.9%)	2/17 (11.8%)
Crude prevalence ratio‡ (95% CI)		1.0 (Reference group)	0.72 (0.23 to 3.15)	1.45 (0.21 to 8.04)
Risk-adjusted† prevalence ratio‡ (95% CI)		1.0 (Reference group)	1.96 (0.18 to 6.31)	3.37 (0.05 to 10.21)
RE-ADMISSION AT 30 DAYS				
Measure		Heparin only group	Heparin to DOAC group	Heparin to warfarin group
Number		37 (17.9% of sample)	153 (73.9% of sample)	17 (8.2% of sample)
Events	All-cause	7/37 (18.9%)	14/153 (9.1%)	2/17 (11.8%)
	VTE-specific	5/37 (13.5%)	2/153 (1.3%)	0/17 (0%)
	Bleeding-related	1/37 (2.7%)	0/153 (0%)	0/17 (0%)
Crude prevalence ratio‡ (95% CI)		1.0 (Reference group)	0.35 (0.16 to 0.86)	0.45 (0.07 to 1.63)
Risk-adjusted† prevalence ratio‡ (95% CI)		1.0 (Reference group)	1.11 (0.65 to 2.27)	2.16 (0.50 to 4.58)

## Discussion

This study retrospectively reviewed the anticoagulation strategies used in a sample of patients with acute PE who had PERT activations and evaluated whether the different types of anticoagulation administered were associated with specific patient outcomes. Patients who were treated with heparin (whether UFH or LMWH) initially and subsequently transitioned to warfarin had a longer hospital LOS with otherwise unchanged overall outcomes, including rates of in-hospital bleeding, all-cause 30-day mortality, and all-cause 30-day re-admissions.

The most common strategy for therapeutic anticoagulation in this study was the initiation of UFH, followed by subsequent transition to a DOAC. This finding was consistent with evidence published over the past decade, which established the safety and efficacy of DOACs for the treatment of acute PE [15]. In our study, the major reasons for preferring warfarin over DOAC included a diagnosis of antiphospholipid antibody syndrome, patient preference, and insurance coverage or affordability issues. In some patients, clinicians preferred to use warfarin because of a diagnosis of malignancy, left ventricular thrombus, and morbid obesity. However, evidence from recent studies suggests that DOACs may be safely used in patients with left ventricular thrombus [16], morbid obesity [17], and malignancy [18]. Thus, the clinician's preference for warfarin in some cases may reflect not following evidence-based guidelines. This observation is also in line with the findings reported by Wheelock and colleagues in 2021 [6], who reviewed US Medicare prescription claims data encompassing 325,666 clinicians (between 2013 to 2018) and reported that 1 in 5 general medicine practitioners continued to use warfarin exclusively for their patients despite an increase in DOAC prescribing for US Medicare patients during the study period. In 2020, the American Society of Hematology recommended the use of DOACs over warfarin to treat venous thromboembolism [19].

A strategy of treating patients with heparin (either UFH or LMWH) initially followed by a transition to warfarin was associated with a longer hospital LOS in this study. This observation was consistent with the findings reported by several prior studies [20-23]. Margolis et al. reviewed hospitalizations for acute PE within MarketScan’s Hospital Drug Database (between November 2012 and December 2013) and found that patients treated with rivaroxaban stayed 1.71 days fewer in the hospital than those treated with warfarin [21]. In another study, Weeda and colleagues (2017) [20] compared the hospital LOS for acute PE patients treated with rivaroxaban versus those bridged to warfarin and found that rivaroxaban use was associated with a 1.4-day reduction in hospital LOS. Similar findings were observed by Saint and colleagues (2017) in a retrospective review of 152 patients with VTE, who noted that the hospital LOS was shorter for patients treated with any DOAC compared to warfarin [23]. The association of longer hospital LOS with warfarin therapy is likely attributable to the need for bridging (overlapping) anticoagulation spanning over a few days and the difficulty in achieving an internationalized normalized ratio within the therapeutic range [24]. In most randomized controlled trials comparing DOACs and warfarin, the time in the therapeutic range for patients receiving warfarin was approximately 70% during the study period [25]. This may be even lower for patients in real-world settings [26], which might explain the longer LOS among patients with acute PE who are bridged to warfarin.

The 30-day mortality rate in our study was not associated with any of the therapeutic anticoagulation strategies described in the study involving either the transition of UFH or LMWH to warfarin or DOAC. While the 30-day mortality rate was higher in the “heparin-only” group, this represented selection bias as patients who had died before being transitioned to any other agent were solely represented in this group. Results from numerous clinical studies published over the past two decades have established the safety and efficacy of DOACs for the treatment of PE. In a meta-analysis of three randomized trials enrolling 386 Japanese patients, Senoo et al. [27] reported that the risk of all-cause mortality was not different between patients treated with warfarin and those treated with DOACs. In another systematic review including 27,127 patients from six randomized trials, Gómez-Outes et al. [28] found that the risk of all-cause mortality was unchanged among patients treated with DOACs compared to “standard” treatment. Overall, the 30-day mortality rate for our entire cohort was 10.6%, which was also comparable to that reported previously in the literature [1-3]. Of note, the majority of the study patients were not at low risk of mortality, where low-risk, intermediate low-risk, intermediate high-risk, and high-risk PE were noted in six (2.9%), 50 (24.1%), 132 (63.8%), and 19 (9.2%) cases, respectively.

In our study, the rates of in-hospital bleeding did not differ between the groups of patients treated with different anticoagulation strategies. Overall, 6.8% of patients experienced bleeding complications, which was similar to the rate of bleeding reported in prior studies of patients with acute PE. The rates of clinically relevant bleeding were 4.3% for apixaban in the AMPLIFY trial [29], 10.3% for rivaroxaban in the EINSTEIN-PE trial [30], and 8.5% for edoxaban in the HOKUSAI-VTE trial [31]. It is important to note here that clinically relevant bleeding events of any severity were recorded in our study. If only major bleeding was considered, the number of events would have been even lower, while the rates could have been higher if trivial bleeding events were also included [32]. For instance, in the RE-COVER trials, the rates of any bleeding were 15.6% and 22.1% for patients in the dabigatran and warfarin arms, respectively [33]. While the safety of DOACs for the treatment of acute PE is well established, some studies also suggest that the risk of major bleeding may be lower with DOACs [33,34]. In a systematic review of six trials, Gómez-Outes and colleagues reported that the risk of major bleeding for DOACs compared to conventional treatment (parenteral anticoagulant followed by a vitamin K antagonist) in patients with acute VTE was lower (relative risk 0.62; absolute risk reduction of 0.6%) [28]. However, this effect was not consistent across all included trials, and there was high heterogeneity amongst the pooled studies [28]. The lack of an association between the type of anticoagulant and bleeding events in our study may be due to the small number of overall bleeding events in the study patients.

The rate of 30-day all-cause re-admission was not significantly associated with the strategy of therapeutic anticoagulation. Due to the small number of events, we did not perform multivariate analysis for cause-specific re-admission rates. Nevertheless, the rates of VTE-related 30-day re-admissions in the “heparin to DOAC” and “heparin to warfarin” groups were 1.3% and 0%, respectively. Moreover, no bleeding-related re-admissions occurred in the “heparin to DOAC” and “heparin to warfarin” groups. These findings did not signal any safety concerns. In 2017, Kohn et al. [35] performed a systematic review of five studies that compared outcomes with rivaroxaban versus warfarin for patients with acute PE and found that the rates of re-admission amongst the two groups were similar. In another meta-analysis of 10 studies including 35,019 patients with acute PE, Gómez-Outes, and colleagues [36] reported that the rates of re-admissions did not differ among patients treated with DOACs and those treated with warfarin. On the other hand, in a real-world analysis of patients from four large databases in the United States, Weycker et al. (2020) [34] compared the outcomes of 20,561 patients treated with apixaban and 35,080 patients treated with warfarin. At a 180-day follow-up period, patients in the apixaban group had a lower risk of major bleeding (adjusted hazard ratio: 0.71), recurrent venous thromboembolism (adjusted hazard ratio: 0.77), as well as clinically relevant non-major bleeding (adjusted hazard ratio: 0.72) [34]. Since we only considered in-hospital bleeding events and 30-day re-admissions, we might not have been able to capture this association in the present study.

The limitations of our study should be kept in mind when interpreting the results. First, the study was non-randomized and retrospective, which implies that there is a risk of confounding bias. However, we performed multivariate regression analysis to adjust for the effect of multiple confounders in an attempt to mitigate confounding bias while assessing the association of different anticoagulation strategies with overall patient outcomes. Second, the number of events of in-hospital bleeding and re-admission was low, particularly in the “heparin to warfarin” group, which may have reduced the power of this study to detect possible associations. However, this aspect of our study is congruent with real-world practices and the tendency of practitioners to relegate warfarin use to a limited number of clinical situations. Lastly, we did not perform any matched analysis in this study, as less than one-tenth of the patients were transitioned to warfarin. The degree of imbalance in the size of the different groups implied that a large proportion of the sample would be discounted in the analysis, which would preclude any meaningful matched analysis. Despite these limitations, our study reflects real-world practices and outcomes for patients with acute PE. Recommended management protocols for patients resulting from randomized clinical trials are rarely replicated in real-world settings, and therefore, this study more closely reflects the outcomes of patients in real-world settings.

## Conclusions

This study retrospectively reviewed the anticoagulation strategies used in a sample of patients with acute PE who had PERT activations and evaluated whether the different types of anticoagulation administered were associated with specific patient outcomes. Patients who were initially treated with heparin (whether UFH or LMWH) and subsequently transitioned to warfarin had a longer hospital LOS when compared to patients who transitioned from heparin to a DOAC or were treated with LMWH alone. However, the rates of in-hospital bleeding, all-cause 30-day mortality, and all-cause 30-day re-admissions were not associated with the strategy of therapeutic anticoagulation employed. These results suggest that a strategy of anticoagulation employing heparin as a bridge to warfarin therapy is less preferable when compared to anticoagulation with heparin alone or transitioning to a DOAC.
